# CSF GABA is reduced in first-episode psychosis and associates to symptom severity

**DOI:** 10.1038/mp.2017.25

**Published:** 2017-03-14

**Authors:** F Orhan, H Fatouros-Bergman, M Goiny, A Malmqvist, F Piehl, Karolinska Schizophrenia Project (KaSP) Consortium, Göran Engberg, Sophie Erhardt, Lilly Schwieler, Funda Orhan, Anna Malmqvist, Mikael Hedberg, Lars Farde, Simon Cervenka, Lena Flyckt, Karin Collste, Pauliina Ikonen, Fredrik Piehl, Ingrid Agartz, S Cervenka, K Collste, P Victorsson, C M Sellgren, L Flyckt, S Erhardt, G Engberg

**Affiliations:** 1 Department of Physiology and Pharmacology, Karolinska Institutet, Stockholm, Sweden; 2 Department of Clinical Neuroscience, Centre for Psychiatry Research, Karolinska Institutet, Stockholm, Sweden; 3 Neuroimmunology Unit, Department of Clinical Neuroscience, Karolinska Institutet, Karolinska University Hospital, Stockholm, Sweden; 4 Stanley Center for Psychiatric Research, Broad Institute of MIT and Harvard, Cambridge, MA, USA

## Abstract

Schizophrenia is characterized by a multiplicity of symptoms arising from almost all domains of mental function. γ-Aminobutyric acid (GABA) is the primary inhibitory neurotransmitter in the brain and is increasingly recognized to have a significant role in the pathophysiology of the disorder. In the present study, cerebrospinal fluid (CSF) concentrations of GABA were analyzed in 41 first-episode psychosis (FEP) patients and 21 age- and sex-matched healthy volunteers by high-performance liquid chromatography. We found lower CSF GABA concentration in FEP patients compared with that in the healthy volunteers, a condition that was unrelated to antipsychotic and/or anxiolytic medication. Moreover, lower CSF GABA levels were associated with total and general score of Positive and Negative Syndrome Scale, illness severity and probably with a poor performance in a test of attention. This study offers clinical *in vivo* evidence for a potential role of GABA in early-stage schizophrenia.

## Introduction

Schizophrenia is characterized by positive and negative symptoms, as well as by cognitive deficits, in particular in domains related to attention and verbal working memory.^1, 2^ For half a century, the dopamine hypothesis has dominated theories regarding the pathophysiology of schizophrenia. However, although many symptoms can be linked to dopaminergic dysregulation, it has been suggested that causative abnormalities may lie elsewhere.^3^ In this regard, focus has been directed to glutamatergic dysregulation and, in particular, to an *N*-methyl-D-aspartate receptor hypofunction.^4, 5, 6^ Thus, patients with schizophrenia show elevated levels of post-mortem brain and cerebrospinal fluid (CSF) kynurenic acid, an endogenous *N*-methyl-D-aspartate receptor antagonist.^7, 8, 9, 10^ Several lines of research have also implicated the inhibitory neurotransmitter γ-aminobutyric acid (GABA) in the pathophysiology of schizophrenia and a number of studies have identified deficits in parvalbumin containing GABA neurons in schizophrenia.^11^ One of the most consistent post-mortem findings in schizophrenia is a decreased expression of the 67 kDa isoform of glutamic acid decarboxylase, a key enzyme in the biosynthesis of GABA.^12, 13, 14, 15^ In line with this, several studies have shown an association between *GAD1*, the gene for the enzyme 67 kDa isoform of glutamic acid decarboxylase, and schizophrenia.^16, 17, 18^ Furthermore, congruent with a reduced expression of 67 kDa isoform of glutamic acid decarboxylase, post-mortem studies reveal lower GABA levels in multiple brain regions including the nucleus accumbens, thalamus, amygdala and hippocampus in patients with schizophrenia.^19, 20, 21, 22, 23^

In contrast to genetic and post-mortem studies, *in vivo* studies of GABA in schizophrenia have been inconclusive. Using proton magnetic resonance spectroscopy (^1^H-), some studies found a decrease,^24, 25, 26^ some an increase,^27, 28^ and yet others found no changes in GABA levels^29, 30, 31^ in patients with schizophrenia. Differences in GABA levels between patients and unaffected controls appear dependent on the brain area investigated, the duration of illness, as well as on the medication.^25, 26, 27, 28, 30, 31^ Recently, a study using positron emission tomography utilizing [^11^C]flumazenil suggested an impaired GABA neurotransmission in patients with schizophrenia, a finding that was also associated with positive symptoms.^32^ Further, several studies analyzing CSF GABA in patients with schizophrenia, most of them performed during the 1980s, have yielded mostly negative and partly inconsistent results.^33, 34, 35, 36, 37, 38, 39, 40, 41^

Taken together, there is an increasing body of evidence from genetic and post-mortem studies implicating an altered GABA transmission as a significant component of schizophrenia pathophysiology. However, robust evidence from CSF studies of an involvement of GABA is still lacking. We here analyze CSF GABA and four other amino acids, that is, glutamate, glycine, taurine and tyrosine, with a sensitive analytical assay, in well-characterized groups of healthy controls and patients with first-episode psychosis (FEP), most of them drug naive to antipsychotic medication. We hypothesize that CSF GABA is reduced in FEP patients, and that low levels of GABA associate to worse symptoms and cognitive deficits.

## Materials and methods

### Subject population

The study was approved by the Regional Ethics Committee in Stockholm and conformed to the tenets of the Declaration of Helsinki. All subjects were included from March 2011 through January 2014, after providing written informed consent. This study formed part of the Karolinska Schizophrenia Project, a multidisciplinary research consortium that investigates the pathophysiology of schizophrenia.

### FEP patients

Forty-one FEP patients (25 male and 16 female) who met the Diagnostic and Statistical Manual of Mental Disorders (DSM-IV) criteria for schizophrenia (*n*=12), schizophreniform disorder (*n*=14), severe depression with psychotic features (*n*=1), delusional disorder (*n*=3), brief psychotic disorder (*n*=1), psychotic disorder not otherwise specified (*n*=9) or schizoaffective syndrome (*n*=1) were recruited from psychiatric emergency wards and 3 psychiatric outpatient clinics in Stockholm. Diagnosis was established based on a structured clinical interview of the DSM-IV or a consensus diagnostic procedure. All patients were re-assessed after approximately 1.5 years and were then found to meet the criteria for the following DSM-IV diagnoses: schizophrenia (*n*=25), psychotic disorder not otherwise specified (*n*=5), delusional disorder (*n*=4), brief psychotic disorder (*n*=1), schizoaffective syndrome (*n*=3) and no diagnosis (*n*=3). Exclusion criteria were neurologic or severe somatic illness, substance abuse and autism spectrum disorder. Absence of major brain abnormalities was confirmed using magnetic resonance imaging. All patients underwent an extensive clinical characterization, including the Global Assessment of Functioning (GAF; where symptom and functioning dimensions were assessed separately), the Positive and Negative Syndrome Scale (PANSS), Clinical Global Impression (CGI), Alcohol Use Disorders Identification Tests and Drug Use Disorders Identification Tests. All patients included in this study were somatically healthy and free from any substance abuse disorder. Tobacco use was permitted and 11 of the 41 patients (27%) used tobacco (smoking or snuff). Occasional medication with sedatives and anxiolytics were allowed during the course of the study. At the time of CSF sampling, 12 out of 41 patients (29%) were treated with benzodiazepines (BZDs). Eighteen out of 41 patients (44%) were under antipsychotic treatment at the time of CSF sampling (mean time (±s.e.m.) 7.2±1.82 days). Patients with more than 1 month of treatment with antipsychotics were not included in the study, with the exception of the inclusion of one patient that had been treated for 57 days. Twelve out of 41 patients were naive to all medications. Antipsychotics used were olanzapine, aripiprazole, risperidone, quetiapine or haloperidol (see Supplementary Table S1). Individual medication was maintained in all patients throughout the test period, although the dosages of anxiolytics/hypnotics may have been slightly adjusted. Duration of untreated psychosis was based on information from the patients or his/her relatives. For most patients (*n*=34), GAF, PANSS, cognitive testing and lumbar puncture were all performed within a 10-day period (mean time (±s.e.m.): 5.5±0.4 days), whereas seven of the patients underwent these investigations during a period from 14 to 40 days (mean time (±s.e.m.): 19.4±3.6 days).

### Healthy control subjects

Twenty-one healthy control subjects (11 males and 10 females) were recruited by advertisement. Medical examination was made by routine laboratory blood and urine tests, physical examination, as well as a brain magnetic resonance imaging examination. The Mini International Neuropsychiatric Interview was used to exclude previous or current psychiatric illness. Further exclusion criteria were previous or current use of illegal drugs and first-degree relatives with psychotic illness. All participants were free from medication and any form of substance abuse evaluated with Alcohol Use Disorders Identification Tests/Drug Use Disorders Identification Tests at the time of the study. None of the subjects had any first-degree relative with a psychiatric diagnose. In all but one case, no structural brain abnormality was detected using magnetic resonance imaging, as evaluated by an experienced neuroradiologist at the MR Centre, Karolinska University Hospital, Solna. This individual exhibited signs of demyelinating disease on magnetic resonance imaging, but did not fulfill criteria for a clinically isolated syndrome or multiple sclerosis,as the clinical neurological exam was normal and there was no history of relevant neurological symptoms. CSF examination revealed oligoclonal bands, but no other abnormalities. Test results were similar to other controls and therefore this subject was not excluded from the analysis. For all healthy controls, cognitive test session and lumbar puncture were all performed within mean time (±s.e.m.): 14.5±3.01 days.

### CSF collection

Efforts were made to reduce confounding factors of the lumbar puncture procedure that could influence analysis of CSF amino acids.^42^ These efforts include the use of a disposable atraumatic needle (22G Sprotte, Geisingen, Germany) that was inserted at the L 4-5 level with all individuals in the right decubitus position. Further, the same volume of CSF (18 ml) was allowed to drip into a plastic test tube, protected from light. CSF supernatant from all subjects was divided into 10 aliquots that were frozen at −80 °C within 1 h of sampling following centrifugation (Sigma 5810R, Eppendorf, Hamburg, Germany at 3500 r.p.m. (1438 *g*) for 10 min) to separate cells and supernatant, respectively. The majority of subjects (*n*=37; 23 patients and 14 controls) underwent the lumbar puncture between 0745 and 2200 h after a night’s sleep. Owing to clinical routines, morning sampling was not possible in the remaining FEP patients (*n*=18). To control for this confounding factor, seven controls also underwent lumbar puncture during the same time interval (that is, 1030 and 1315 h). All subjects were instructed to avoid physical activity during the preceding 8 h; however, it was not feasible to monitor rest or posture in this regard. Importantly, no correlation between CSF GABA levels and the point of time for lumbar puncture was observed (Pearson; all: *r*=−0.14, *P*=0.28; controls: *r*=−0.06, *P*=0.81; patients: *r*=−0.17, *P*=0.29). This is in analogy with BenMenachem *et al.*,^43^ showing no differences in CSF GABA in healthy controls between lumbar puncture in the afternoon and next morning sampling.

A fresh sample was analyzed for cell numbers, albumin, immunoglobulin G content and the presence of immunoglobulin G and immunoglobulin M antibodies to Borrelia, as well as with immune electrophoresis.

### Analysis of CSF GABA

Samples were subsequently analyzed for GABA (and additional amino acids, that is, glutamate, taurine, glycine and tyrosine) with a gradient elution reversed-phase high pressure liquid chromatography system, including a gradient pump (Spectra System P4000, Waltham, MA, USA), a degasser (Spectra System SCM 400), a Luna 100 C18(2) column (50 × 2 mm i.d., 5 μm particle size, Phenomenex, Torrance, CA, USA) and a fluorescence detector (Jasco FP-920, Tokyo, Japan) operating at excitation and emission wavelengths of 344 and 495 nm, respectively. The chromatographic separation was performed at room temperature (22 °C). CSF from FEP patients and healthy controls were derivatized for 60 s at room temperature with *O*-phthaldialdehyde/2-mercaptoethanol reagent. The reagent was prepared by dissolving 27 mg *O*-phthaldialdehyde in 0.5 ml ethanol (99.5%), 4.5 ml borate buffer (0.4 M boric acid adjusted to pH 10.4 with sodium hydroxide) and 20 μl 2-mercaptoethanol was added.

Detection of amino acid gradients was performed with two degassed mixture mobile phases. Mobile phase A consists of 0.04 M sodium acetate buffer (pH 6.95) containing 2.5% (v/v) of methanol, 2.5% (v/v) of tetrahydrofuran and mobile phase B consists of methanol. The flow rate of the mobile phase was 0.7 ml min^−1^ throughout the analysis and all gradient changes were linear. The gradient conditions were as follows: initial conditions are 100% mobile phase A; from time 0 to 11 min the gradient changes to 70% mobile phase A and 30% mobile phase B; from 11 to 13 min the gradient changes to 10% mobile phase A and 90% mobile phase B; from 14 min the gradient changes to 100% mobile phase A and remains in this condition until the next injection. Samples of 20 μl were manually injected into the system. The signals from the fluorescence detector were transferred to a computer and analyzed by Datalys Azur Software (Grenoble, France). Approximate retention time of GABA was 9.8 min (glutamate 1.2 min; taurine 5.5 min; tyrosine 11.5 min).

### Cognitive testing

The Measurement and Treatment Research to Improve Cognition in Schizophrenia Consensus Cognitive Battery^44^ was used to evaluate cognitive function. This battery captures key cognitive domains relevant to schizophrenia. The Measurement and Treatment Research to Improve Cognition in Schizophrenia Consensus Cognitive Battery includes 10 tests that measure 7 cognitive domains: Speed of processing (Brief Assessment of Cognition in Schizophrenia: Symbol Coding, Category Fluency: Animal Naming, Trail Making Test: Part A); Attention/vigilance (Continuous Performance Test-Identical Pairs); Working memory (Wechsler Memory Scale-3rd Edition: Spatial Span, Letter-Number Span); Verbal learning (Hopkins Verbal Learning Test-Revised); Visual learning (Brief Visuospatial Memory Test-Revised); Reasoning and problem solving (Neuropsychological Assessment Battery: Mazes) and Social cognition (Mayer–Salovey–Caruso Emotional Intelligence Test: Managing Emotions). One psychologist (HFB) administered all the tests.

### Statistical analysis

The normality of data was determined using D’Agostino and Pearson’s omnibus normality test. One-tailed tests of significance (Mann–Whitney *U*-test) were performed in the comparison between CSF GABA levels in FEP patients and healthy controls, and in the correlation analyses as a directional change, that is, decreased GABA levels in FEP patients, could be hypothesized at this stage. Two-tailed tests were performed to determine the possible effects of various medications on CSF GABA (Table 2), as the direction of any change in CSF GABA levels could not be anticipated. To assess the relative importance of potential confounders, we used binary logistic regression or *χ*^2^-test, as well as the R package ‘relaimpo’.^45^ Here individual regressor’s contribution to a multiple linear regression model is quantified using six different methods. Although the different methods produced similar results, we here report an approach based on sequential *R*^2^s that takes care of the dependence on orderings by averaging over orderings using simple unweighted averages (lmg). Comparisons of cognition between FEP patients and controls were analyzed using the unpaired *t*-test with equal s.d. Reported correlation coefficients are Pearson’s *r*. Bonferroni correction was used in the comparison of different cognitive tests between healthy controls and FEP patients, giving an *α*-threshold of 0.005 (0.05/10). Only those cognitive tests that remained significant after the Bonferroni correction was used for the correlation with CSF GABA in FEP patients giving an *α*-threshold of 0.0083 (0.05/6). With regard to the correlation studies between CSF GABA and cognitive tests in healthy controls, the *α*-threshold was set to 0.005 (0.05/10). Symptom ratings were highly correlated (see Supplementary Table S2) and therefore not corrected for repeated measure. To confirm the association between CSF GABA and clinical symptoms, we applied a principal component analysis (Supplementary Information). All analyses were performed using Prism version 6.0 (GraphPad Software, La Jolla, CA, USA), SPSS Statistics version 20.0 (IBM, Armonk, NY, USA), or R statistics (R Development Core Team, Vienna, Austria). Statistical significance was considered when *P*<0.05.

## Results

### Participants

Clinical and demographic characteristics of participants are presented in Table 1. There was no significant difference in age, gender or body mass index between patients and healthy controls. Duration of untreated psychosis was 10.5±1.88 (mean±s.e.m.) months and total PANSS score was 73.9±3.42 (mean±s.e.m.). Eighteen patients (44%) received antipsychotic medication, 12 patients (29%) BZDs, 10 patients (24%) zopiclone, 5 patients (12%) antidepressants and 11 patients (27%) received phenothiazine derivatives. Twenty-nine patients (71%) received a combination of some of these drugs at the time of CSF sampling. Twelve out of 41 patients (29%) were naive to all medications (see Supplementary Table S1).

### CSF GABA in FEP patients versus healthy controls

**Figure 1:**
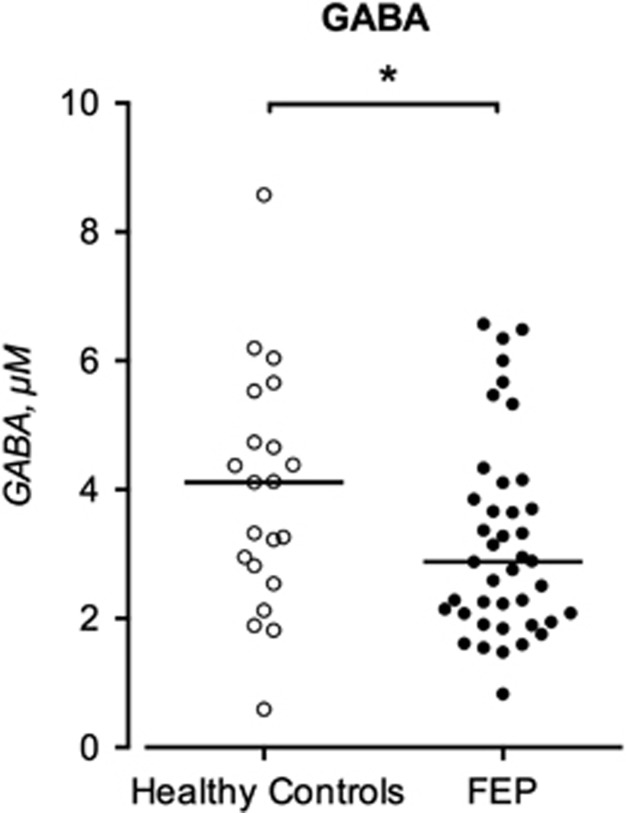
GABA in the CSF of healthy controls (HC, *n*=21) and (FEP, *n*=41) patients. Each point represents the concentration of a single CSF sample and the horizontal lines represent the median for each group. Statistical differences between controls and FEP patients were determined using Mann–Whitney *U*-test. *P<0.05. CSF, cerebrospinal fluid; FEP, first-episode psychosis; GABA, γ-aminobutyric acid.

The CSF levels of GABA in FEP patients and healthy controls are displayed in Figure 1.

The CSF GABA concentration was significantly lower in FEP patients compared with healthy controls (median 2.88 μM, interquartile range 2.02–6.57 μM, *n*=41 vs median 4.11 μM, interquartile range 2.68–5.13 μM, *n*=21, *P*=0.042). No significant associations were found between CSF GABA levels and age, gender, body mass index or tobacco use (see Supplementary Table S3). CSF GABA levels did not differ between FEP patients that were naive to antipsychotic treatment and those on antipsychotic treatment (*P*=0.85). Neither did CSF GABA levels differ between treated and untreated FEP patients with regard to BZDs (*P*=0.30), zopiclone (*P*=0.47), antidepressants (*P*=0.37), phenothiazine derivatives (*P*=0.62) or all drugs combined (*P*=0.15) (see Table 2). To further investigate the impact of potential confounders, an R package relaimpo was also made assessing the relative importance of the regressors diagnosis (yes/no), medication (yes/no), age, gender, body mass index and tobacco use (yes/no) in a linear model predicting CSF GABA levels. All metrics allocated the largest share of *R*^2^ to case status (sequential *R*^2^s approach with averaging over orderings using simple unweighted averages: 45%) followed by age (25%), whereas the remaining predictors accounted for 15% or below (Supplementary Figure S1). Given the wide bootstrap confidence intervals, together with a previous study showing an age effect on GABA levels, we adjusted the original analysis for age, but the significant association between case status and CSF GABA levels remained (*P*=0.047).

We also analyzed additional amino acids, that is, glutamate, taurine, glycine and tyrosine, in our samples. The CSF concentration of these was not significantly different between patients and healthy controls (Supplementary Figure S2). Further, the glutamate/GABA ratio was not significantly different between patients and controls (*P*=0.16).

### Cognitive performance in FEP patients versus healthy controls and correlations between cognitive performance and CSF GABA in FEP patients

Compared with healthy controls, FEP patients showed a reduction in performance in all cognitive domains tested, the most salient related to attention, speed of processing and visual/verbal learning. Six of the cognitive domains remained significant after Bonferroni correction (Table 3). Moreover, a positive correlation was found between CSF GABA and scores of the attention/vigilance test Continuous Performance Test-Identical Pairs performance in FEP patients (*r*=0.37, *P*=0.01; which remained borderline significant following the Bonferroni correction (significance threshold of *P*<0.0083) (Table 4). No associations were found between CSF GABA and other cognitive domains tested. In healthy controls, correlations between CSF GABA and cognition were also observed with regard to the cognitive domains visual learning (*r*=−0.44, *P*=0.02) and social cognition (*r*=−0.59, *P*=0.002). Only the correlation with social cognition remained after Bonferroni correction (threshold of *P*<0.005).

### Correlations between CSF GABA and symptoms in FEP patients

Symptoms among patients were profiled using PANSS, CGI and GAF. We observed negative correlations between CSF GABA and total score on PANSS (*r*=−0.30, *P*=0.03) in FEP patients (Table 4). For the general psychopathology subscale of PANSS, we found a similar correlation (*r*=−0.31, *P*=0.02). Correlation analyses using the subscale measuring positive symptoms reached a nearly significant association (*r*=−0.26, *P*=0.0503), whereas the subscale negative symptoms did not (*r*=−0.19, *P*=0.12). Moreover, CSF GABA was found to correlate positively to scores of the symptom dimension of GAF (*r*=0.31, *P*=0.02). With regard to scores of the functioning dimension of GAF a trend towards statistical significance was observed (*r*=0.24, *P*=0.06). A negative correlation between CSF GABA and CGI scores (*r*=−0.38, *P*=0.007) was detected.

Notably, ratings for PANSS, GAF and CGI scales were highly correlated (see Supplementary Table S2), making a Bonferroni correction for repeated measures inappropriate. To overcome the problem with adequate control of type I and II errors, a principal component analysis was performed (for details, see Supplementary Information) followed by linear regression modeling of extracted individual principal component scores. This confirmed an association between symptoms and low CSF GABA levels (*β*=−0,27; *P*=0.020; Table 4).

## Discussion

The present study shows a reduction in CSF GABA levels in patients with FEP compared with healthy controls. Furthermore, CSF GABA concentration, which was unrelated to antipsychotic and/or anxiolytic medication, was found to correlate with general and total score of PANSS, as well as to illness severity, such that lower CSF GABA levels predicted higher symptom levels.

Mounting clinical and experimental data suggest a role of GABA in the pathophysiology of schizophrenia (*cf*. Introduction). However, evidence for this is mainly indirect and analyses of GABA in CSF of patients with schizophrenia have failed to give a conclusive result in this regard. Although Van Kammen *et al.*^41^ found significantly lower CSF GABA levels in young women with schizophrenia, most previous studies analyzing GABA in the CSF from patients with schizophrenia have failed to observe any difference compared with controls.^33, 34, 35, 36, 37, 38, 39, 40^ These studies, all performed in the 70s or 80s, have been limited by the lack of a control group of age-matched healthy volunteers or by the sensitivity of the GABA assay (enzymetric fluorometric method, ion-exchange column chromatography or radio-receptor assay). Thus, discrepancies between present data, utilizing a well-characterized cohort, healthy volunteers as controls and top-of-the-art high-performance liquid chromatography analysis of GABA and previous literature may be explained by differences in study design and methods of GABA analysis.

The present finding of lower CSF GABA levels in FEP patients likely reflects a reduced overall GABAergic neurotransmission in the brain. The observed direction of changes is in line with previous post-mortem studies showing a reduced GABA synthesis in schizophrenia (*cf*. Introduction). Further, in excellent agreement with present findings, a recent positron emission tomography study, utilizing the GABA-A receptor ligand [^11^C]flumazenil, indicate an impaired GABA transmission in the orbital frontal cortex in patients with schizophrenia.^32^ Our findings also reveal negative correlations between CSF GABA and total and general PANSS, such that low CSF GABA levels predicted high general severity of illness. In addition, low CSF GABA concentrations were associated with reduced scores of the symptom and functioning dimensions of GAF, as well as with high CGI scores, indicating that symptoms and illness severity associate with lower levels of CSF GABA. In line with a large body of studies, investigating cognitive functions in schizophrenia, FEP patients showed a significant reduction in performance compared with healthy controls in all parts of our cognitive test battery (Table 3). Moreover, CSF GABA levels were found to be positively correlated with Continuous Performance Test-Identical Pairs (although it did not fully meet the Bonferroni-corrected significance threshold of *P*<0.008), a cognitive test that measures attention, a cognitive domain impaired in patients with schizophrenia.^2, 46, 47^ A relationship between CSF GABA and cognitive performance is in line with a recent magnetic resonance spectroscopy study showing that GABA predicts working memory.^24^ Notably, in contrast to patients, healthy controls showed a negative correlation between CSF GABA and performance in the social cognition test. This finding is in line with several clinical reports showing that increased GABA transmission, induced by BZDs or vigabatrin (an inhibitor of GABA transaminase, thereby increasing GABA levels throughout the brain) is associated with cognitive impairments.^48, 49, 50^

A strength of the present study is that the majority of the patients were drug naive with respect to antipsychotics at the time of the lumbar puncture. No differences in CSF GABA levels were observed between patients on treatment and those without antipsychotic treatment (Table 2), indicating that low CSF GABA levels are not a result of antipsychotic treatment. Owing to a lack of power in this subgroup analysis, we cannot fully exclude drug effects in this regard. However, the observation that antipsychotics do not lower brain GABA levels is in line with an magnetic resonance spectroscopy study investigating patients with a 6-month treatment with atypical antipsychotics.^31^ Furthermore, increased CSF GABA levels were observed following 30 days of treatment with sulpiride as well as following treatment with different neuroleptics for many years in long-stay hospitalized patients with schizophrenia.^36^ In addition, Gattaz *et al.*^51^observed no change in free CSF GABA levels in patients with schizophrenia after three months of haloperidol treatment. Altogether, it seems implausible that the lower levels of CSF GABA seen in FEP patients are a consequence of antipsychotic treatment.

A limitation of the present study is the fact that 29% of the FEP patients were on BZDs, drugs well known to primarily affect GABAergic neurotransmission. However, previous studies have shown that intravenous injection of diazepam to neurological patients is associated with an increase in CSF GABA levels^52^ and, in consonance, administration of diazepam to rats and mice results in increased brain GABA concentrations.^53, 54^ Taken together, given previous results on the effects of BZDs or antipsychotic drugs on brain GABA concentrations it appears unlikely to be that the presently observed reduction in CSF GABA levels in FEP is the result of such medications. This is strengthened by the fact that CSF GABA levels did not differ between the drug-naïve patients and those patients that were on BZD treatment at the time of lumbar puncture (Table 2) and by the fact that our extended bootstrap linear modeling did not indicate medications as an important co-factor.

A question of importance is if the lower CSF GABA in patients with schizophrenia contributes to the pathophysiology and specific symptoms of the disease. BZDs, known to increase GABAergic transmission via a modulatory action at the GABA-A receptor, are frequently used as adjunctive medication in first-episode patients. It is generally accepted that these drugs are primarily used for anxiolysis and do not affect positive or negative symptoms of the disease. Although a few studies report some favorable effects of BZDs on psychotic symptoms,^55^ recent meta-analysis give no evidence for antipsychotic efficacy of additional benzodiazepine medication.^56^ Further, administration of GABA-A receptor antagonists is not typically associated with psychotomimetic symptoms. Similarly, clinical trials with GABA-B receptor agonists, such as baclofen or γ-hydroxybutyric acid, have given no unanimous picture for antipsychotic effects of these drugs.^57, 58^ Indeed, the most common adverse effects observed of vigabatrin is behavioral disturbances, ranging from irritability and confusion to psychotic reactions.^59^ Thus, the many experimental and post-mortem studies, suggesting a role of GABA in the pathophysiology of schizophrenia is not supported by the clinical experience of medication with GABA receptor agonists. Although CSF GABA correlated to PANSS scores, illness severity and attention, it remains to be evaluated whether the reduction in CSF GABA is primarily involved in the generation of positive/negative symptoms.

For decades, studies have implicated a reduced GABAergic transmission as part of the pathophysiology of schizophrenia. To the best of our knowledge, the present study is the first to show that FEP patients display low CSF GABA levels and that this condition is associated with the severity of illness, psychotic symptoms and probably attentional deficits.

## Figures and Tables

**Table 1:** Demographic and clinical characteristics of the study population

*Characteristic*	*Mean*±*s.e.m. (*n)^a^		P*-value*
	*Healthy controls (*n=*21)*	*FEP patients (*n=*41)*	
Age (years)	25.9±1.11 (21)	29.2±1.06 (41)	0.06^b^
Gender (male/female)	11/10	25/16	0.52^c^
BMI (kg/m^2^)	22.2±0.55 (20)	23.1±0.62 (39)	0.33^b^
% Tobacco users	0%	27%	—
DUP (months)	—	10.5±1.88 (37)	—
Days of antipsychotic treatment	—	7.2±1.82 (18)	—
			
*Medication*			
Antipsychotics	0%	44%	—
Benzodiazepines	0%	29%	—
Zopiclone	0%	24%	—
Antidepressants	0%	12%	—
Phenothiazine derivatives	0%	27%	—
			
*PANSS*			
Positive	—	19.5±0.86 (41)	—
Negative	—	15.9±1.21 (41)	—
General	—	38.5±1.87 (41)	—
Total	—	73.9±3.42 (41)	—
			
*Level of functioning*			
GAF symptoms	—	35.6±1.99 (41)	—
GAF functioning	—	46.2±2.17 (41)	—
CGI score	—	4.4±0.17 (41)	—

Abbreviations: BMI, body mass index; CGI, Clinical Global Impression; DUP, duration of untreated psychosis; FEP, first-episode psychosis; GAF, Global Assessment of Functioning; PANSS, Positive and Negative Syndrome Scale.

a Unless otherwise indicated.

b Binary Logistic regression.

c *χ*^2^-test.

**Table 2:** CSF GABA levels (μM) with regard to medication

*Medication*	*Patient on drug*	*Patient off drug*	P*-value*^a^
	*CSF GABA* *Mean±s.e.m. (*n)	*CSF GABA* *Mean±s.e.m. (*n)	
Antipsychotics^b^	3.1±0.29 (18)	3.2±0.37 (23)	0.85
Benzodiazepines^c^	2.8±0.25 (12)	3.4±0.32 (29)	0.30
Zopiclone	3.5±0.42 (10)	3.1±0.29 (31)	0.47
Antidepressants^d^	2.6±0.48 (5)	3.3±0.26 (36)	0.37
Phenothiazine derivatives^e^	3.0±0.31 (11)	3.3±0.31 (30)	0.62
Any treatment^f^	3.0±0.20 (29)	3.7±0.60 (12)	0.15

Abbreviations: CSF, cerebrospinal fluid; GABA, γ-aminobutyric acid.

a Unpaired *t*-test with equal s.d.

b All antipsychotics combined.

c All benzodiazepines combined.

d All antidepressants combined.

e All phenothiazine derivatives combined.

f Either antipsychotics, benzodiazepines, zopiclone, antidepressants and phenothiazine derivatives or a combination of these. Patients off drug were all drug naive.

**Table 3:** Comparison of different cognitive tests between healthy controls and FEP patients

*Test*	*Cognitive domain*	*Mean±s.e.m.*		P*-value*^a^
		*Healthy Controls (*n=*21)*	*FEP patients (*n=*40)*	
CPT-IP	Attention/vigilance	3.0±0.08	2.19 ±0.11	<0.0001^b^
TMT	Speed of processing	23.2±1.13	32.8±2.13	0.003^b^
BACS-SC	Speed of processing	61.2±1.18	46.0±1.87	<0.0001^b^
Fluency	Speed of processing	25.2±1.28	21.6±0.90	0.02
WMS-III	Working memory (non-verbal)	18.5±0.60	15.9±0.50	0.003^b^
LNS	Working memory (verbal)	15.5±0.55	13.3±0.50	0.008
NAB: MAZES	Reasoning and problem solving	22.9±0.89	19.1±0.88	0.007
BVMT-R	Visual learning	29.5±1.10	22.4±1.10	<0.0001^b^
MSCEIT	Social cognition	98.0±1.23	89.9±2.0	0.007
HVLT-R	Verbal learning	28.7±0.65	24.0±0.73	<0.0001^b^

Abbreviations: BACS-SC, Brief Assessment of Cognition in Schizophrenia Symbol Coding; BVMT-R, Brief Visuospatial Memory Test-Revised; CPT-IP, Continuous Performance Test-Identical Pairs; FEP, first-episode psychosis; HVLT-R, Hopkins Verbal Learning Test-Revised; LNS, Letter-Number Span; MSCEIT, Mayer–Salovey–Caruso Emotional Intelligence Test; NAB: MAZES, Neuropsychological Assessment Battery: Mazes; TMT, Trail Making Test; WMS-III, Wechsler Memory Scale-3rd Edition.

a Unpaired *t*-test with equal s.d.

b Significant after Bonferroni-corrrection, *α*-value=0.005.

**Table 4:** Correlations between CSF GABA, clinical symptoms and cognitive performance in FEP patients

	r	P*-value*^a^
*PANSS*		
Positive	−0.26	0.05
Negative	−0.19	0.12
General	−0.31	0.02^b^
Total	−0.30	0.03^b^
		
*Severity of illness*		
GAF symptom dimension	0.31	0.02
GAF functioning dimension	0.24	0.06
CGI score	−0.38	0.007
		
*Cognitive tests*		
CPT-IP	0.37	0.01^c^
TMT	−0.038	0.41
BACS-SC	−0.028	0.43
WMS-III	0.016	0.46
BVMT-R	−0.095	0.28
HVLT-R	−0.12	0.24

Abbreviations: BACS-SC, Brief Assessment of Cognition in Schizophrenia Symbol Coding; BVMT-R, Brief Visuospatial Memory Test-Revised; CGI, Clinical Global Impression; CPT-IP, Continuous Performance Test-Identical Pairs; CSF, cerebrospinal fluid; FEP, first-episode psychosis; GABA, γ-aminobutyric acid; GAF, Global Assessment of Functioning; HVLT-R, Hopkins Verbal Learning Test-Revised; PANSS, Positive and Negative Syndrome Scale; TMT, Trail Making Test; WMS-III, Wechsler Memory Scale-3rd Edition.

a Pearson’s correlation.

b Significant also after principal component analysis.

c Bonferroni-corrrected, *α*-value=0.0083.
