# Supplementary material for: CSF GABA is reduced in first-episode psychosis and associates to symptom severity
Source: Mol Psychiatry. 2017 Mar 14;23(5):1244–50. doi: 10.1038/mp.2017.25 (PMC5984082; doi:10.1038/mp.2017.25)
Supplement: Supplementary Information [file mp201725x1.docx]

**Supplementary information**

**Full title**

**CSF GABA is reduced in first-episode psychosis and associates to symptom severity**

**Authors**

Funda Orhan, MSc.^1^, Helena Fatouros-Bergman, PhD.^2^, Michel Goiny, PhD.^1^, Anna Malmqvist, MD.^1^, Fredrik Piehl, MD., PhD.^3^, Karolinska Schizophrenia Project (KaSP) consortium^4^, Simon Cervenka, MD., PhD.^2^, Karin Collste MD.^2^, Pauliina Victorsson, MD. ^2^, Carl M Sellgren, MD, PhD^1,5^ , Lena Flyckt, MD., PhD. ^2^, Sophie Erhardt, PhD.^1^ and Göran Engberg, PhD.^1^

**Author information**

**Affiliations:** ^1^ Dept. of Physiology and Pharmacology, Karolinska Institutet, Stockholm, Sweden. ^2^ Dept. of Clinical Neuroscience, Centre for Psychiatry Research, Karolinska Institutet, Stockholm, Sweden. ^3^ Dept. of Clinical Neuroscience, Neuroimmunology Unit, Karolinska Institutet, Karolinska University Hospital, Stockholm, Sweden ^4^Members of Karolinska Schizophrenia Project (KaSP) are listed at the end of the article, ^5^ Stanley Center for Psychiatric Research, Broad Institute of MIT and Harvard, Cambridge, MA, USA

**Principal component analysis**

To further analyze the association between CSF GABA and clinical symptoms we applied a principal component analysis (PCA) to log transformed ratings (‘prcomp’ function in R; ‘center’ and ‘scale’ equal to ‘TRUE’). Individual principal component scores were then extracted and linear regressions against CSF GABA were performed.

*Correlations between CSF GABA and symptoms ratings in FEP patients*

Symptoms among patients were profiled using PANSS, CGI, and GAF. Ratings for these scales, as well as subscales (PANSS and GAF), were highly correlated. To study associations between symptom profile and CSF GABA levels with adequate control of type I and II errors a principal component analysis was performed. Total sum on PANSS loaded highest on the first principal component that explained 60 % of variability and using the first 4 principal components 90 % of the variability could be explained. We then extracted individual loadings on the first principal components and performed a simple linear regression against CSF GABA levels. This analysis suggested an association between high loading on first component, i.e., high symptom scores, and low CSF GABA levels (β= -0,27; p=0.020). Adding the forthcoming three components (loading foremost on GAF and ratings for negative symptoms on PANSS) the association remained similar (β= -0,27; p=0.019) while none of the other components associated with CSF GABA. Performing simple linear regressions for each of the total sum on PANSS, and each subscale (PANSS general, positive, and negative) suggested significant associations for the two PANSS scales loading highest on the first component (PANSS total; β= -0.30, p= 0.03, and PANSS general; β= -0.31, p= 0.02), while the subscale measuring positive symptoms (β= -0.26, p= 0.0503) and negative symptoms (β= -0.19, p= 0.12) did not reach significance.

**Supplementary Tables**

Table S1: Medication details

|  | MEDICATION (drugs given within 15 h prior to lumbar puncture) | | | |
| --- | --- | --- | --- | --- |
| Patient | Antipsychotics (mg) | Benzodiazepines or Zopiclone (mg) | Phenothiazine derivatives (mg) | Antidepressants (mg) |
| 1 | — | — | — | — |
| 2 | — | — | — | — |
| 3 | — | Oxazepam (5) | — | — |
| 4 | — | — | — | — |
| 5 | — | — | — | — |
| 6 | — | Zolpidem (10) | Propiomazine (25) | — |
| 7 | — | — | — | — |
| 8 | — | Oxazepam (15) | Propiomazine (25) | — |
| 9 | — | — | — | Citalopram (20) |
| 10 | — | — | — | — |
| 11 | — | — | — | — |
| 12 | — | Zopiclone (7.5) | — | — |
| 13 | — | — | — | — |
| 14 | — | Oxazepam (15),  Zopiclone (7.5) | Propiomazine (25) | — |
| 15 | — | Oxazepam (15),  Zopiclone (7.5) | Propiomazine (25) | — |
| 16 | — | Zopiclone (7.5) | Propiomazine (50) | — |
| 17 | — | — | — | — |
| 18 | Aripriprazole (10) | Zopiclone (7.5) | Propiomazine (25) | — |
| 19 | — | Oxazepam (10),  Zopiclone (7.5) | — | Paroxetine (20) |
| 20 | — | Nitrazepam (5) | — | — |
| 21 | — | — | — | — |
| 22 | — | — | — | — |
| 23 | — | — | — | — |
| 24 | Quetiapine (100) | — | — | Sertraline (50) |
| 25 | — | Zopiclone (7.5) | — | — |
| 26 | Olanzapine (10) | — | Propiomazine (25) | — |
| 27 | Aripriprazole (10) | — | — | — |
| 28 | Olanzapine (10),  Quetiapine (300) | Oxazepam (5-10),  Nitrazepam (5),  Zopiclone (7.5-15) | Propiomazine (25) | — |
| 29 | Olanzapine (15) | — | — | — |
| 30 | Olanzapine (10),  Quetiapine (600) | Oxazepam (10) | Propiomazine (25),  Alimemazine (60) | — |
| 31 | Haloperidol (1),  Olanzapine (15) | — | — | — |
| 32 | Olanzapine (15) | Diazepam (10),  Zopiclone (7.5) | — | Mirtazapine (30) |
| 33 | Risperidone (4) | Oxazepam (10) | — | Mirtazapine (30) |
| 34 | Risperidone (2) | — | — | — |
| 35 | Olanzapine (20) | — | Propiomazine (25) | — |
| 36 | Olanzapine (30) | — | — | — |
| 37 | Olanzapine (10) | — | — | — |
| 38 | Olanzapine (25) | — | Alimemazine (80) | — |
| 39 | Risperidone (4) | — | — | — |
| 40 | Risperidone (4) | Oxazepam (10),  Zopiclone (7.5) | — | — |
| 41 | Olanzapine (15) | — | — | — |

Table S2: Correlation of symptoms in FEP patients assessed by GAF, PANSS and CGI


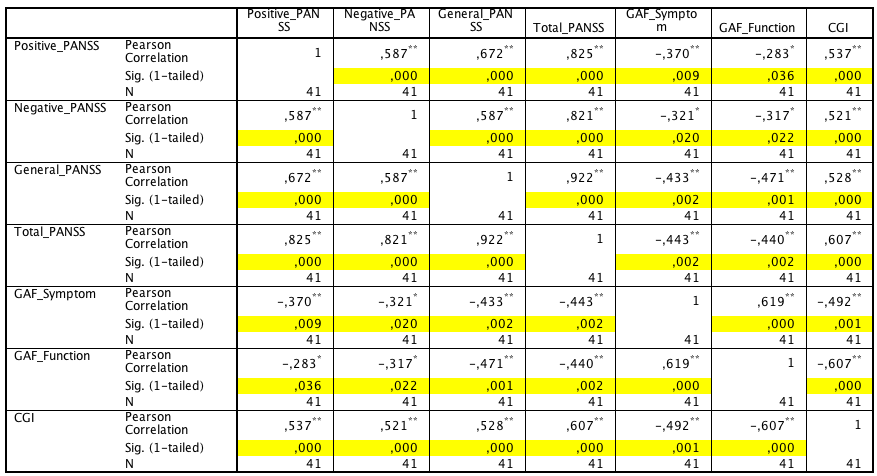
(screenshot from SPSS)

Table S3. Clinical characteristics and their association with CSF GABA in FEP patients

| Characteristics | r | P-value^1^ |
| --- | --- | --- |
| Age (years) | 0.07 | 0.65 |
| Gender (male/female) | -0.08 | 0.61 |
| BMI (kg m^-2^) | -0.09 | 0.56 |
| Tobacco use | 0.11 | 0.48 |
| ^1^Pearson Correlation  Abbreviations: BMI = Body Mass Index | | |

**
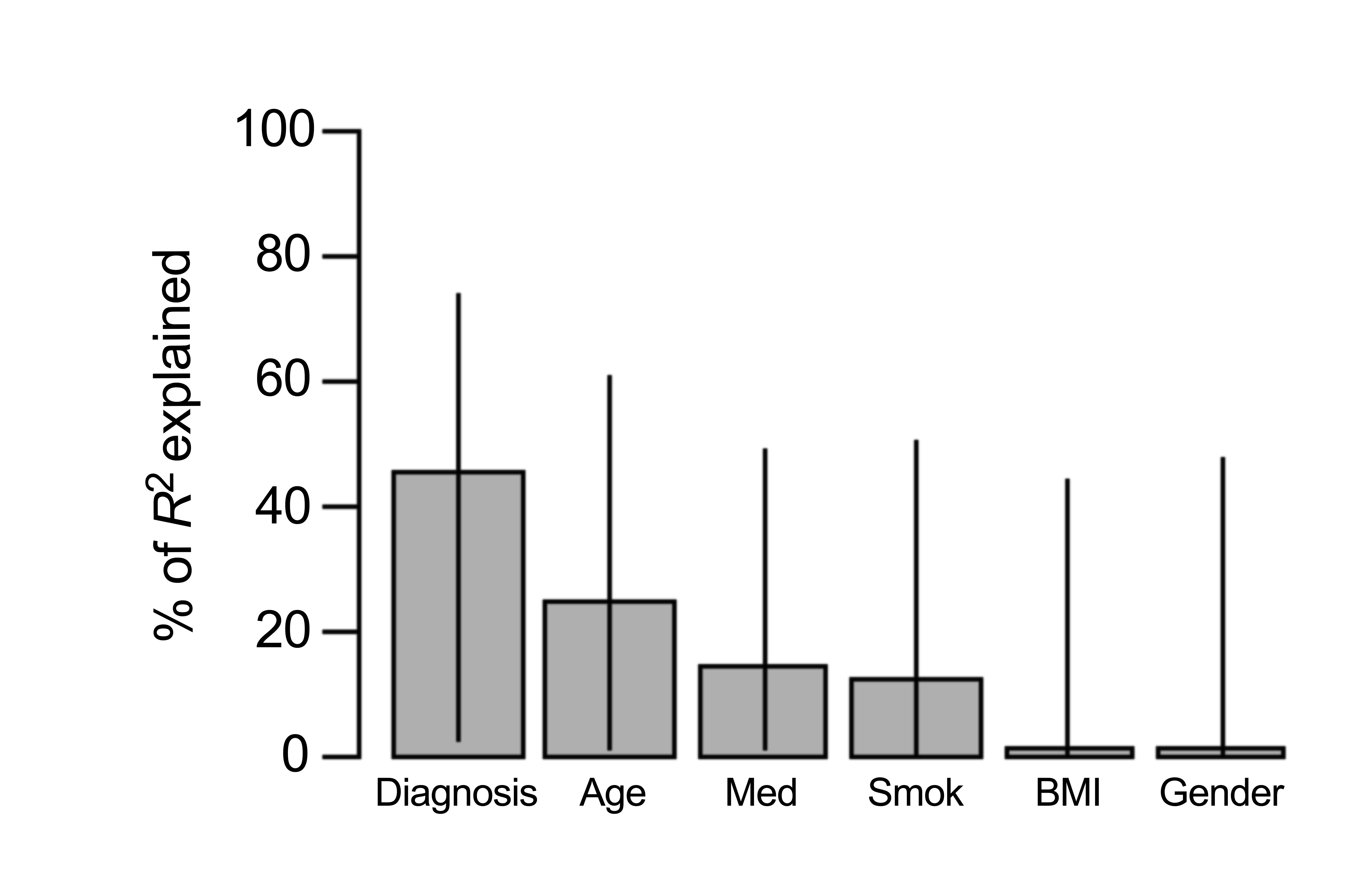
Supplementary Figures**

Figure S1: Relative importance of case status and potential confounders for cerebrospinal fluid (CSF) levels of GABA in a cohort consisting of 41 first-episode psychosis patients and 21 healthy controls. Analysis was performed using the R script ‘relaimpo’. The metrics ‘lmg’ is displayed in the figure using a method based on *R^2^* partitioned by averaging over orders. Y-axis is normalized to the sum 100. Med = medication (y/n), Smok = smoking (y/n), BMI = body mass index. Bars represent 95 % bootstrap confidence intervals.

Figure S2: (A) glutamate, (B) glycine, (C) taurine and (D) tyrosine in the cerebrospinal fluid (CSF) of healthy controls and FEP patients. Each point represents the concentration of a single CSF sample, and the horizontal lines represent the median for each group (Mann-Whitney U test). With regard to glycine, one healthy control and one FEP patient were considered outliers (Grubbs test) and therefore excluded.
